# Comparative transcriptome analyses of immune responses to LPS in peripheral blood mononuclear cells from the giant panda, human, mouse, and monkey

**DOI:** 10.3389/fgene.2022.1053655

**Published:** 2023-01-06

**Authors:** Shun Li, Caiwu Li, Lixiang Chen, Hua Yang, Xiaonan Ren, Chunhua Xu, Bin Wu, Chao Wang, Yun Ling, Yinzhong Shen, Hongzhou Lu, Weiping Liu, Xiaohui Zhou

**Affiliations:** ^1^ Department of Animal Model, Shanghai Public Health Clinical Center, Fudan University, Shanghai, China; ^2^ Key Laboratory of State Forestry and Grassland Administration on Conservation Biology of Rare Animals in the Giant Panda National Park, China Conservation and Research Center for the Giant Panda, Dujiangyan, Sichuan, China; ^3^ Department of Infectious Disease, Shanghai Public Health Clinical Center, Fudan University, Shanghai, China; ^4^ Department of Infectious Diseases and Immunology, Shanghai Public Health Clinical Center, Fudan University, Shanghai, China; ^5^ Shenzhen Key Laboratory of Pathogen and Immunity, National Clinical Research Center for infectious disease, State Key Discipline of Infectious Disease, The Third People’s Hospital of Shenzhen, Second Hospital Affiliated to Southern University of Science and Technology, Shenzhen, Guangdong, China

**Keywords:** LPS, immune responses, comparative transcriptome analyses, PBMCs, giant panda

## Abstract

Gram-negative bacteria are major pathogens that can cause illnesses in giant pandas. Lipopolysaccharides (LPS), components of Gram-negative bacteria, can activate immune responses in mammals (i.e., humans and mice) through recognition by toll-like receptors (TLRs). However, the giant pandas’ immune response to LPS stimulation and the differences between the giant panda and other mammals are not fully known. In this study, we administrated peripheral blood mononuclear cells (PBMCs) from giant pandas, humans, C57BL/6 mice, and rhesus monkeys by LPS treatment at 6 h followed by RNA sequencing (RNA-seq), respectively, with control of non-stimulation. KEGG analyses of differentially expressed genes (DEGs) pathways indicated that LPS could activate the classic signaling pathway of NF-κB in PBMCs from those four tested species. Thus, similar to the other three species, NF-κB is an LPS-responsive regulator of innate immune responses in giant pandas. Furthermore, the expression patterns of adapter genes, inflammatory cytokine genes, chemokines, interferon genes, cytokine genes related to cell growth and development, costimulatory molecules, Th1/Th2 cytokine genes, Th17 cytokine genes, Th9, and Th22 cytokine genes were compared among giant pandas and three other species. Our data indicated that in addition to the similar expression patterns of certain genes among giant pandas and other species, the unique expression pattern response to LPS in giant pandas was also discovered. Furthermore, Th9, Th17, and Th22 cells might be involved in the response to LPS in giant pandas at this tested time point. This study reveals that LPS-induced immune responses have different sensitivities and response timelines in giant pandas compared with other mammals. This study facilitates further understanding of the role of the TLR signaling pathway and the immune system in giant pandas, which might be helpful for disease prevention and protection.

## Introduction

The giant panda (*Ailuropoda melanoleuca*) is a world-famous endangered mammal species native to China. Similar to other mammals, giant pandas suffer from a range of diseases, including infectious diseases, which seriously threaten them, especially captive populations (Zhao et al., 2017; Ji et al., 2022). The immune system is pivotal both in fighting against infections and in the pathogenesis of various diseases (Block et al., 2022). Thus, deeply exploring and understanding the immunity of giant pandas is important for disease prevention and protection. Compared with the limited accessibility of disease-relevant tissues, profiling blood samples is a minimally invasive way to assess and analyze immune health and function (Sen et al., 2017; Schmiedel et al., 2018; Pessini et al., 2020). Moreover, blood serves as a pipeline for circulating immune cells throughout the whole body and has the ability to reflect the signatures of diseases or immune dysfunction (Banchereau et al., 2016; Kotliarov et al., 2020). Currently, the characteristics of immunity in humans and commonly used laboratory animals (i.e., mice and monkeys) are widely and frequently studied (Bosteels et al., 2020; Liu et al., 2020; Netea et al., 2020; Brueggeman et al., 2022). However, very little is known about the characteristics of immunity in giant panda blood and how that immunity might be similar to or different from immunity in other mammals.

Peripheral blood mononuclear cells (PBMCs) are a critical part of the immune system used to fight infection (Li et al., 2021). PBMCs were frequently applied as an *ex vivo* model to characterize cytokine gene expression after stimulation by purified TLR (toll-like receptor) ligands or live probiotics (Slawinska et al., 2021; Lee et al., 2022). Lipopolysaccharide (LPS) is an important pathogenic component present in the cell walls of Gram-negative bacteria (Tartey and Takeuchi, 2017), and Osamu et al. (Takeuchi et al., 1999) reported that TLR2 and TLR4 were found to be involved in LPS-mediated signaling. However, TLR4 but not TLR2 plays a critical role in LPS signaling (Takeuchi et al., 1999), and LPS recognition is predominantly mediated by TLR4 (Poltorak et al., 1998). TLR4 belongs to a pattern recognition receptor (PRR), and it mainly expresses in macrophages, dendritic cells, eosinophilic granulocytes, and mastocytes (Cagliero et al., 2022; Jing et al., 2022). LPS can be recognized by TLR4 and activates the signaling pathway related to nuclear factor-kappa B (NF-κB) to promote the expression of cytokine genes and the release of cytokines in immune cells (Khan et al., 2022; Martino et al., 2022). During this process, myeloid differentiation primary response 88 (MyD88) and TIR-domain-containing adapter-inducing interferon-β (TRIF) serve as two main adapter proteins that transmit the signal generated by extracellular TLR4 into the cells (Tartey and Takeuchi, 2017). Thus, TLR4 plays an important role both in the formation of the innate immune response and in affecting the initiation of the adaptive immune response.

Several *in vitro* and *in vivo* studies about LPS-stimulated PBMCs were performed on different animals (e.g., chickens and lambs) followed by qRT-PCR to understand the changes of certain gene expressions (Aksel and Akyüz, 2021; Slawinska et al., 2021). However, the number of tested genes was limited. RNA-seq has been widely applied for analyzing global gene expression levels, and it could be used for transcriptome analyses of the gene expression changes after such cellular responses to uncover functional differences and/or phenotypes in PBMCs among different species.

Because the giant panda genome had already been reported (Li et al., 2010), previous studies of the giant panda transcriptome have mainly focused on mRNA levels from tissues or whole blood (Li et al., 2020; Shen et al., 2022; Zheng et al., 2022). However, how the genes changed in PBMCs of giant pandas after LPS stimulation and what are the similarities and differences of transcriptional profile compared with other mammals are still unknown.

In this study, we aimed to compare the changes and expression profiles of related genes after LPS stimulation *in vitro* on giant pandas, humans, mice, and monkeys. In order to achieve this purpose, LPS was separately administered in PBMCs from giant pandas, humans, C57BL/6 mice, and rhesus monkeys for 6 h. The RNA-seq was used for constructing the transcriptional profiles of each species, and the expression of some inflammatory- or interferon-related or other immune genes was further analyzed and compared among the four species. Taken together, our study could offer a new angle of comparative medicine view for further understanding the function of TLRs in the immune system and the roles of stimuli in activating the innate and adaptive immune system in giant pandas and other species. Those data may contribute to facilitating further immunological investigations for giant pandas aimed at developing strategies for their disease prevention and protection.

## Materials and methods

### Ethics statement and sample preparation

Ethical approval was obtained from the Research Ethics Committee of Shanghai Public Health Clinical Center under permission numbers 2022-A005-01 and 2022-S016-02. Samples (5 ml whole blood) were taken from three giant pandas in Shanghai Wild Animal Park, separately. Blood (5 ml) was collected from three healthy rhesus monkeys housed in the animal facility in Shanghai Public Health Clinical Center, Fudan University (Shanghai, China). Due to the limited amount of blood in mice, samples were collected from twenty 6–8 -week-old C57BL/6 mice housed under specific pathogen-free conditions at the animal facilities in the Shanghai Public Health Clinical Center. Around 100–150 µL blood samples were collected from each mouse at each time, and all the mouse blood was pooled for further experiments. Three healthy volunteers were recruited from the Shanghai Public Health Clinical Center, and 5 ml blood samples were collected from each person.

### Sequence alignment and analysis

Amino acids sequences of TLR4 from giant pandas, humans, mice, and rhesus monkeys were searched and downloaded from the NCBI GenBank database (https://www.ncbi.nlm.nih.gov/gene/?term=). The conserved domains of giant panda TLR4 protein were analyzed by NCBI Conserved Domains (https://www.ncbi.nlm.nih.gov/cdd) (Marchler-Bauer et al., 2017). The transmembrane region of the giant panda TLR4 protein was predicted using TMHMM Server v.2.0 (http://www.cbs.dtu.dk/services/TMHMM/) (Krogh et al., 2001). SWISS-MODEL was used for constructing the 3D crystal structure of giant panda TLR4 protein (https://swissmodel.expasy.org/) (Biasini et al., 2014). Multiple alignments of the amino acid sequence were performed by the NCBI Multiple Alignment Tool (https://www.ncbi.nlm.nih.gov/tools/cobalt/) (Papadopoulos and Agarwala, 2007).

### PBMC isolation and stimulation

All of the blood samples were processed for PBMC isolation using the Ficoll-Hypaque density solution according to a previous study (Li et al., 2021). The cell viability of each sample exceeded 80% by measuring with 0.4% trypan blue. The numbers of PBMCs were calculated, and 1 × 10^6^ cells were pooled into 96-well plates. On the day of stimulation, a volume of 100 µL LPS with a concentration of 2.5 μg/ml was added to the well and mixed gently by pipetting (Slawinska et al., 2021). The cells in the control wells were mock-stimulated with the culture media. The cells were cultured at 37°C and 5% CO_2_, and the cells were harvested at 6 h post-stimulation.

### RNA isolation, library construction, and Illumina sequencing

Approximately 1 × 10^6^ cells of each sample were used for total RNA isolation using the RNA mini kit (Qiagen, Germany). The concentration of RNA was measured by Qubit (Thermo, Waltham, MA, United States), and the quality of RNA was examined by agarose gel electrophoresis. The high-quality RNA was used for RNA-seq library construction using the TruSeq RNA Library Prep Kit V2. The libraries were then pooled and sequenced on NovaSeq 6000 (Illumina) at a depth of approximately 30M reads per sample, and the 150 bp paired-end reads were generated for further analyses.

### Quality control and reference genome alignment

To ensure the quality of sequencing data can be used for downstream analysis, Skewer (v0.2.2) software was used to analyze and remove the sequence reads with adapter and low-quality fragments. FastQC software (v0.11.5) was used for quality control analysis of the preprocessed data and calculating the base ratio of Q20 and Q30. Bowtie2 software was used to align the preprocessed sequence of each sample with the reference genome sequence of the corresponding sequenced species. The reference genomes of the giant panda, human, mouse and monkey were Ailuropoda_melanoleuca genome ASM200744v2, human genome hg38, mouse genome mm10, and Macaca_mulatta genome Mmul_10, respectively.

### Quantification of overall gene expression levels

StringTie software was used to count the read numbers and map reads to genes of all samples. The expression level of each gene was calculated using fragments per kilobase of exon per million mapped fragments (FPKM). FPKM is currently the most commonly used quantitative expression index in paired-end transcriptome sequencing that takes into account the effects of gene length and sequencing data volume. The FPKM calculation formula is total fragments dividing mapped reads (millions) and exon length (KB). The exon length of a gene was defined as the sum of the lengths of the exon non-redundant regions of known transcripts within the gene. Mapped reads were defined as the total number of sequence pairs aligned to the reference genome sequence. Generally, if the FPKM value of a gene is greater than 0.1, the gene is thought to be expressed in the sample.

### Differentially expressed gene analyses

The DESeq2 v1.16.1 package in R software was used for differentially expressed gene screening between the groups of LPS-stimulated and -unstimulated PBMCs. DESeq2 v1.16.1 provides statistical algorithms for determining differential gene expression from gene expression data through the model based on a negative binomial distribution. The *p* values were adjusted by Benjamini and Hochberg’s correction for controlling the false discovery rate). Genes with a corrected *p*-value less than 0.05 and log2FoldChange more than 1 screened by DESeq2 v1.16.1 were defined as differentially expressed genes (DEGs).

### GO and KEGG enrichment analysis for DEGs

For DEGs, Gene Ontology (GO) was analyzed by using topGO (http://www.bioconductor.org/packages/release/bioc/html/topGO.html), and the website (http://www.genome.jp) was used to analyze the KEGG pathway. GO terms and KEGG pathways with an adjusted *p*-value less than 0.05 were considered significantly enriched by DEGs.

### Protein–protein interaction network of immune-related genes

A protein–protein interaction network analysis of immune-related genes was constructed using the STRING web server (https://string-db.org) version 11.0. The network plot was drawn by Cytoscape 3.9.1, and the gene with the highest number of degrees was regarded as a hub gene.

## Results

### Alignment and analysis of giant panda TLR-4 amino acid sequences among different species

**FIGURE 1:**
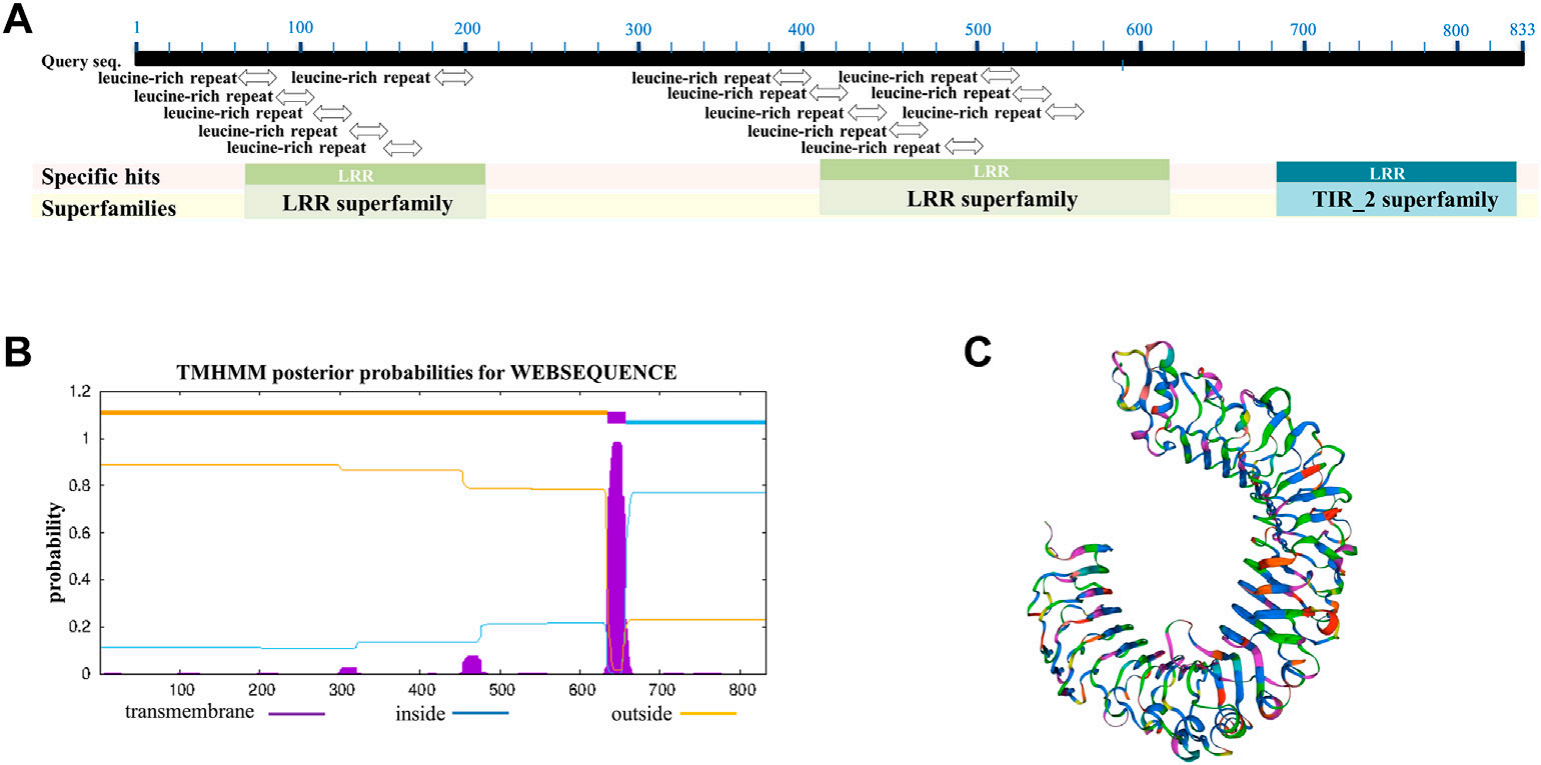
Alignment and analysis of TLR-4 amino acid sequences among different species. **(A)** Conserved domains of giant panda TLR4 protein analysis, showing it contains the conserved domains of LRRs (leucine-rich repeats) and TIR (toll/interleukin 1-receptor). **(B)** Bioinformatic prediction of the transmembrane regions for giant panda TLR4 protein. **(C)** Three-dimensional crystal structure of giant panda TLR4 protein constructed by SWISS-MODEL.

The giant panda toll-like receptor 4 isoform X1 (XP_002929935.3) contains 833 amino acids and was used for multiple sequence alignment and further analysis. The classification of giant panda TLR4 protein belongs to the toll/interleukin-1 receptor domain-containing protein, which has the conserved domains of LRRs (leucine-rich repeats) and TIR (toll/interleukin 1-receptor) (Figure 1A). The prediction of the transmembrane region of giant panda TLR4 showed that the region of amino acids 1–634 were outside cells, amino acids 635–657 belong to the transmembrane domain, and amino acids 658–833 were inside (Figure 1B). The 3D crystal structure of giant panda TLR4 was generated by SWISS-MODEL and showed similarity with the reported crystal structure of human TLR4 (Ohto et al., 2012) (Figure 1C). Multiple sequence alignment revealed that the amino acids of giant panda TLR-4 exhibited 77.29% similarity with human TLR-4 (NP_612564.1), 65.53% similarity with mouse TLR-4 (NP_067272.1), and 77.58% similarity with monkey TLR-4 (XP_014972446.2), respectively (Supplementary Figure S1).

### Transcriptomic profiles of LPS-stimulated and LPS-unstimulated PBMCs from giant pandas

**FIGURE 2:**
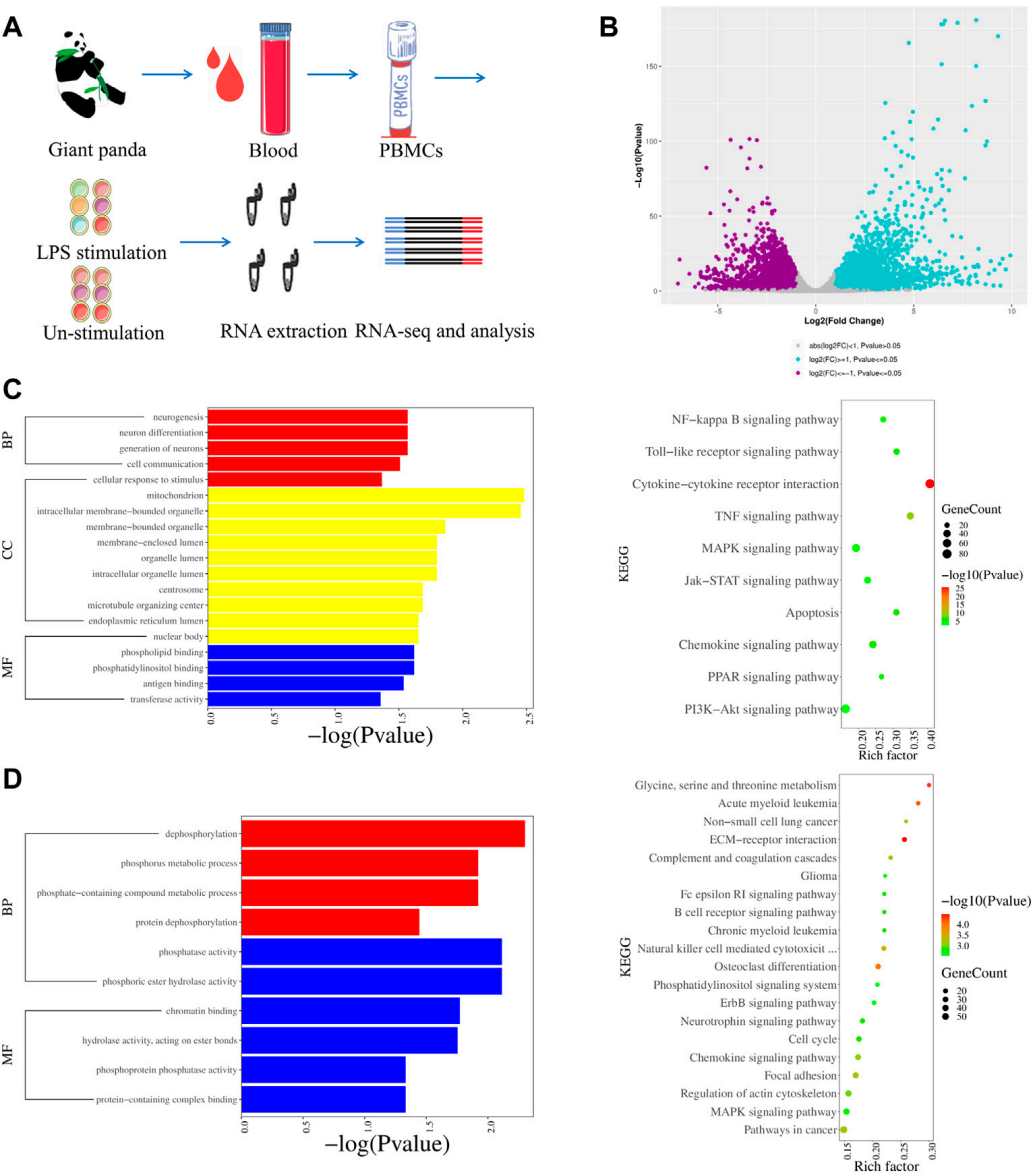
Transcriptomic profiles of LPS-stimulated and LPS-unstimulated PBMCs from giant pandas by RNA-seq. **(A)** Flow diagram of the experimental design. **(B)** Volcano plot shows the DEGs by comparing the LPS-stimulated PBMCs and LPS-unstimulated PBMCs from giant pandas at the time point of 6 h. Significantly differentially expressed genes were defined by both *p*-value (less than and equal to 0.05) and fold change (greater than and equal to 2). **(C)** GO and KEGG enrichments of upregulated DEGs for the group of LPS-stimulated PBMCs vs. LPS-unstimulated PBMCs from giant pandas by RNA-seq. **(D)** GO and KEGG enrichments of downregulated DEGs for the group of LPS-stimulated PBMCs vs. LPS-unstimulated PBMCs from giant pandas by RNA-seq.

We analyzed DEGs by comparing the LPS-stimulated PBMCs (S-P-1, S-P-2, and S-P-3) and the LPS-unstimulated control group (P-1, P-2, and P-3) from giant pandas (Figure 2A). In total, there were 7167 DEGs, including 3973 upregulated and 3194 downregulated genes (Figure 2B). The Gene Ontology (GO) enrichment and KEGG pathways were further analyzed for up- and downregulated genes, respectively. GO analysis revealed that the genes associated with cell communication and cellular response to stimulus were upregulated in LPS-stimulated PBMCs of the giant panda compared with the LPS-unstimulated control cells (Figure 2C). In addition, the genes related to dephosphorylation and phosphorus metabolic processes were downregulated in LPS-stimulated PBMCs of the giant panda compared with the control group (Figure 2D). KEGG analysis revealed that the upregulated genes were mainly enriched into the toll-like receptor signaling pathway, the NF-κB signaling pathway, and the cytokine–cytokine receptor interaction in LPS-stimulated PBMCs of the giant panda by comparing with the LPS-unstimulated PBMCs (Figure 2C). KEGG analysis revealed that the downregulated genes were mainly gathered into the ECM–receptor interaction and the chemokine signaling pathway in the LPS-stimulated PBMC (Figure 2D).

### Transcriptomic profiles of LPS-stimulated and -unstimulated PBMCs from humans, mice, and monkeys

**FIGURE 3:**
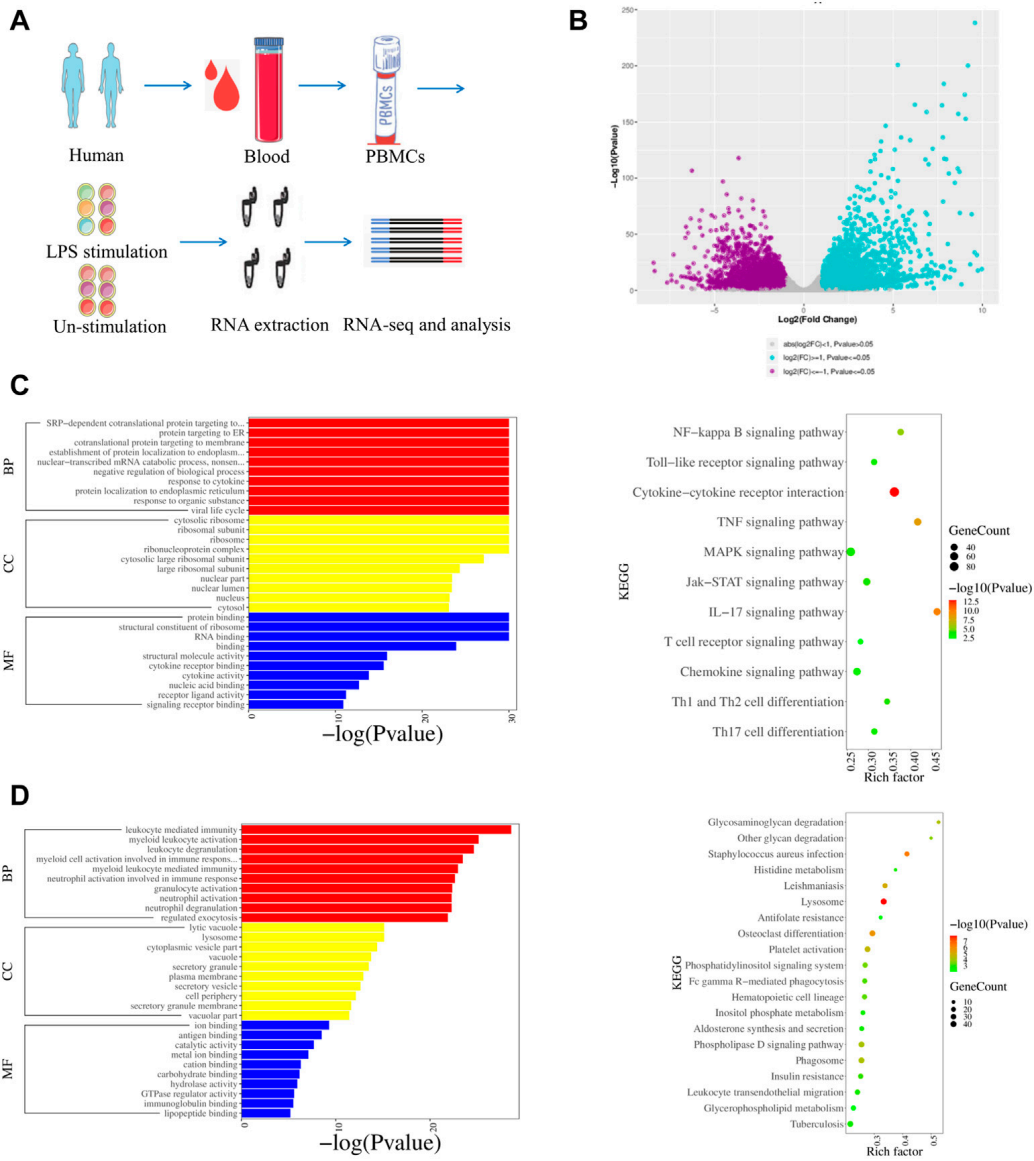
Transcriptomic profiles of LPS stimulated and LPS-unstimulated PBMCs from humans by RNA-seq. **(A)** Flow diagram of the experimental design. **(B)** Volcano plot shows the DEGs by comparing the LPS-stimulated PBMCs and LPS-unstimulated PBMCs from humans at the time point of 6 h. Significantly differentially expressed genes were defined by both *p*-value (less than and equal to 0.05) and fold change (greater than and equal to 2). **(C)** GO and KEGG enrichments of upregulated DEGs for the group of LPS-stimulated PBMCs vs. LPS-unstimulated PBMCs from humans by RNA-seq. **(D)** GO and KEGG enrichments of downregulated DEGs for the group of LPS-stimulated PBMCs vs. LPS-unstimulated PBMCs from humans by RNA-seq.

The gene expression of LPS-stimulated PBMCs from humans (S-H-1, S-H-2, and S-H-3), mice (S-MOU-1, S-MOU-2, and S-MOU-3), monkeys (S-M-1, S-M-2, and S-M-3) and their counterparts in the LPS-unstimulated control groups from humans (H-1, H-2, and H-3), mice (MOU-1, MOU-2, and MOU-3), and monkeys (M-1, M-2, and M-3) were analyzed (Figure 3A; Supplementary Figures S2A, 3A). By comparing the human LPS-stimulated PBMCs (S-H-1, S-H-2, and S-H-3) and the LPS-unstimulated controls (H-1, H-2, and H-3), 8881 DEGs were identified. Among these, 4270 upregulated genes and 4611 downregulated genes were identified (Figure 3B). GO analysis showed that the upregulated genes were mainly associated with response to cytokines, response to organic substances, and other immune system processes (Figure 3C). In addition, KEGG analysis showed that the upregulated genes were involved in the NF-κB signaling pathway, the IL-17 signaling pathway, and the TNF signaling pathway in the LPS-stimulated human PBMCs (Figure 3C). The GO and KEGG analysis for downregulated DEGs in LPS-stimulated human PBMCs revealed that the genes were mainly associated with myeloid leukocyte activation, cell activation, immune system processes, lysosomes, and ECM–receptor interactions (Figure 3D).

The murine DEGs were screened by comparing the group of LPS-stimulated PBMCs (S-MOU-1, S-MOU-2, and S-MOU-3) and LPS-unstimulated counterparts (MOU-1, MOU-2, and MOU-3). In total, 7054 DEGs, including 2625 upregulated genes and 4429 downregulated genes, were identified (Supplementary Figure S2B). Those upregulated genes in the group of LPS-stimulated mouse PBMCs were mainly related to ribosome biogenesis, gene expression, and cellular metabolic processes by GO analysis (Supplementary Figure S2C). KEGG analysis showed that they were involved in the NF-κB signaling pathway, the IL-17 signaling pathway, and the TNF signaling pathway (Supplementary Figure S2C). In addition, these downregulated DEGs were mainly related to immune system processes, immune response, cell activation, and Th1 and Th2 cell differentiation (Supplementary Figure S2D). A comparison of LPS-stimulated PBMCs (S-M-1, S-M-2, and S-M-3) and LPS-unstimulated cells (M-1, M-2, and M-3) from monkeys identified 3311 DEGs, including 1259 upregulated genes and 2052 downregulated genes (Supplementary Figure S3B). GO analysis showed that the upregulated genes were associated with the protein metabolic processes and primary metabolic processes (Supplementary Figure S3C). KEGG analysis indicated that those genes are involved in the NF-κB signaling pathway and the TNF signaling pathway (Supplementary Figure S3C). Compared with non-stimulated PBMCs from monkeys, the downregulated DEGs in LPS-stimulated PBMCs were associated with an integral component of membrane and ECM–receptor interaction by GO and KEGG analysis (Supplementary Figure S3D).

### Comparative analysis of transcriptomic profiles of LPS-stimulated and LPS-unstimulated PBMCs from four species

**FIGURE 4:**
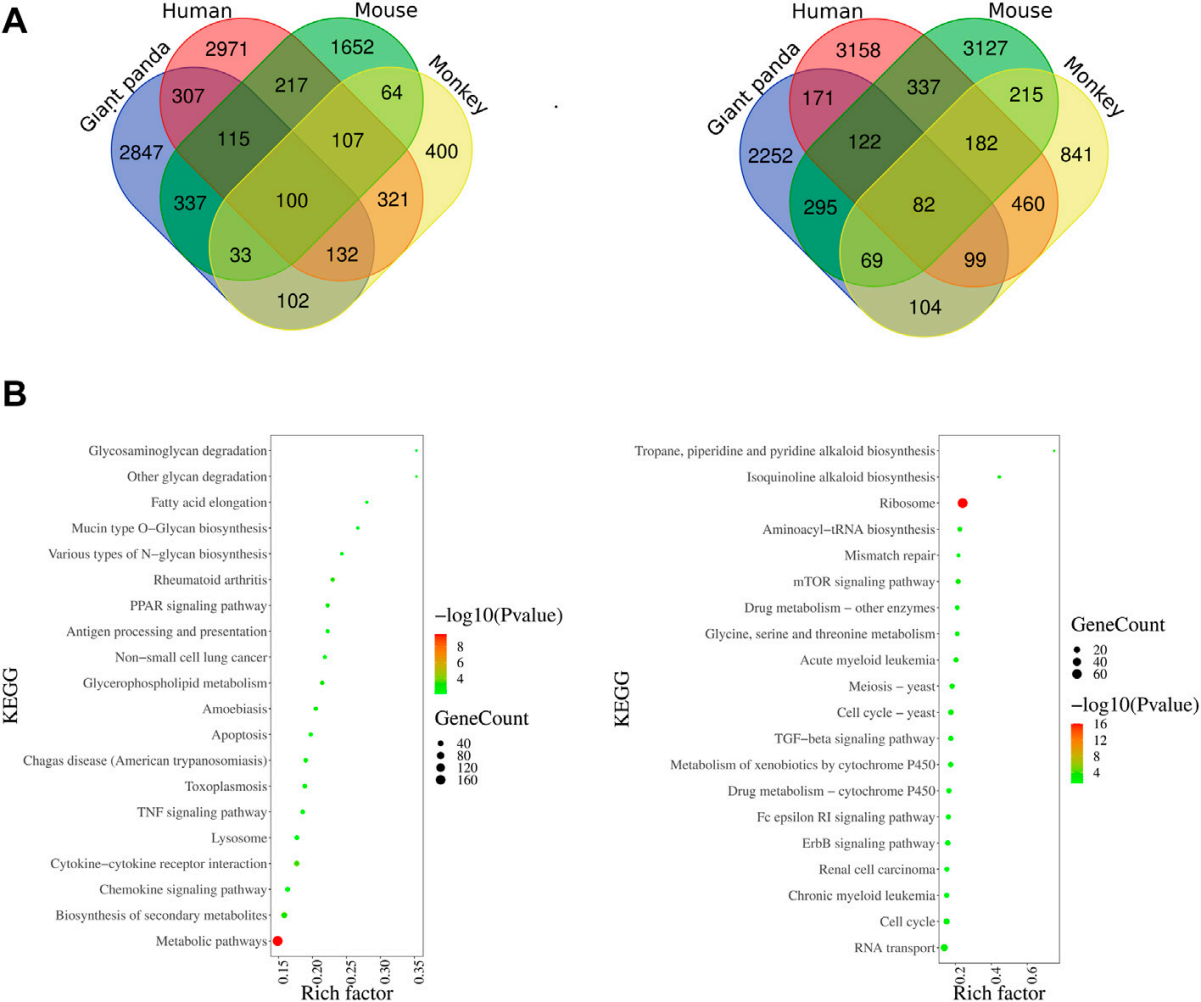
Venn diagram of DEGs among four species and KEGG enrichments of giant panda’s unique DEGs. **(A)** Overlapping upregulated and downregulated DEGs among giant pandas, humans, mice, and monkeys. **(B)** KEGG enrichments of giant panda’s unique upregulated and downregulated DEGs.

The transcriptomic profiles of the LPS-stimulated and -unstimulated PBMCs from giant pandas, humans, mice, and monkeys were comparatively analyzed. One hundred significantly co-upregulated genes and 82 significantly co-downregulated genes were identified in all of the four species examined (Figure 4A). Moreover, 2847 and 2252 genes showed unique upregulated and downregulated trends of expression in giant pandas, when compared with human, mouse and monkey genes (Figure 4A). KEGG analysis revealed that the 2847 upregulated genes were mainly enriched into the TNF signaling pathway and metabolic pathways, and the 2252 downregulated genes were mainly gathered into ribosome pathways (Figure 4B).

LPS recognition is predominantly mediated by TLR4, and then the adapter proteins Myd88 and TRIF transmit the signal into the immune cells to promote the expression of inflammatory cytokine genes and Type I interferon genes. First, we observed the *NFKB1* gene was consistently significantly upregulated in all four species. The significantly upregulated gene *NFKB2* was detected in giant pandas, humans, and mice, but it was not found in the monkey RNA-seq data. The gene expression of MYD88 showed significantly increased expression in giant pandas but was significantly downregulated in humans. In monkeys and mice, MYD88 exhibited non-significant differences in expression. The *IRF3* gene showed non-significantly different expression in the four tested species.

Regarding the inflammatory cytokine genes, IL-1A and IL-1B were significantly upregulated in all four species. IL-6 and TNF had significantly high expression in giant pandas, humans, and mice, but a different pattern was observed in monkeys with a non-significantly increased expression of IL-6 and an undetectable TNF expression level. IL-18 exhibited significantly decreased expression both in giant pandas and mice, but no significant difference in humans and monkeys. In giant pandas, IL-33 was significantly upregulated but showed non-significantly different expression in humans, monkeys, and mice. The chemokines analysis indicated that CCL22 was consistently significantly upregulated in all four species. CXCL8 was significantly upregulated in giant pandas, humans, and monkeys but was undetected in mice. Although CCL2 and CCL3 expression was not found in the giant panda, CCL3 was significantly highly expressed in humans, monkeys, and mice. In addition, CCL2 showed significant upregulation in humans, non-significantly increased expression in monkeys, and significantly decreased expression in mice.

Expression analysis of interferon genes revealed that both IFNG and IFNB1 were significantly upregulated in giant pandas and humans. IFNG was significantly downregulated in mice and showed no significant difference in monkeys. IFNB1 exhibited no significant difference in mice and monkeys. ISG15 was significantly upregulated in giant pandas, humans, and monkeys; however, it was significantly downregulated in mice.

In regard to the cytokine genes with the function of cell growth and development, IL-5, which acts as a growth and differentiation factor for both B cells and eosinophils, exhibited no significant difference in giant pandas, monkeys, and mice, while it showed slightly increased expression in humans with a *p*-value less than 0.05. IL-7, which plays an important role in B- and T-cell development, showed significantly upregulated expression in giant pandas, humans, and monkeys, but it displayed significantly downregulated expression in mice. IL-11, which is related to stimulating the T-cell-dependent development of immunoglobulin-producing B cells, showed no significantly different expression between the LPS-stimulated group and the control counterpart in giant pandas, monkeys and mice. However, it exhibited significantly increased expression in human PBMCs after LPS stimulation.

Costimulatory molecule analysis showed that CD80 and CD40 were consistently significantly upregulated in all four species. CD86 exhibited non-significantly different expression among giant pandas, humans, and monkeys, while it showed significantly increased expression in mice. In addition, CD40LG showed non-significantly differential expression among giant pandas, humans, and monkeys, while it showed significantly decreased expression in mice.

The expression changes of Th1/Th2 cytokine genes were also analyzed. First, the expression of Th1 cytokine IL-2 was undetectable in giant pandas, while it showed non-significant differential expression in humans and monkeys. In mice, IL-2 showed non-significantly decreased expression. The Th2 cytokine IL-3 was not detected in giant pandas, while it showed non-significant differential expression in humans and monkeys. In mice, IL-3 was not found in the gene list in our study. In addition, IL-4 displayed no obvious differential expression pattern in giant pandas, humans, and monkeys, but it displayed significantly decreased expression in mice. The expression patterns of Th1/Th2 regulators were analyzed. IL-10 was consistently significantly upregulated in all four species. IL-12A and IL-12B were significantly upregulated in giant panda and human PBMCs after LPS stimulation. In monkeys, IL-12B showed significantly increased expression, while IL-12A was non-significantly upregulated. In mice, however, IL-12A was significantly downregulated, and IL-12B was non-significantly upregulated.

Second, we analyzed the gene expression of Th17 cytokine genes. IL-17A was significantly upregulated in giant pandas, but it exhibited no significantly different expression when the LPS-stimulated and non-stimulated PBMCs were compared in monkeys and mice. IL-17B was not detected in giant pandas and mice; however, it showed increased expression in humans and non-significant differential expression in monkeys. IL-17C exhibited non-significant differential expression in giant pandas, humans and mice and was not detected in monkeys. IL-23A exhibited non-significant differential expression in giant pandas and mice, while it showed significantly increased expression both in humans and monkeys.

**FIGURE 5:**
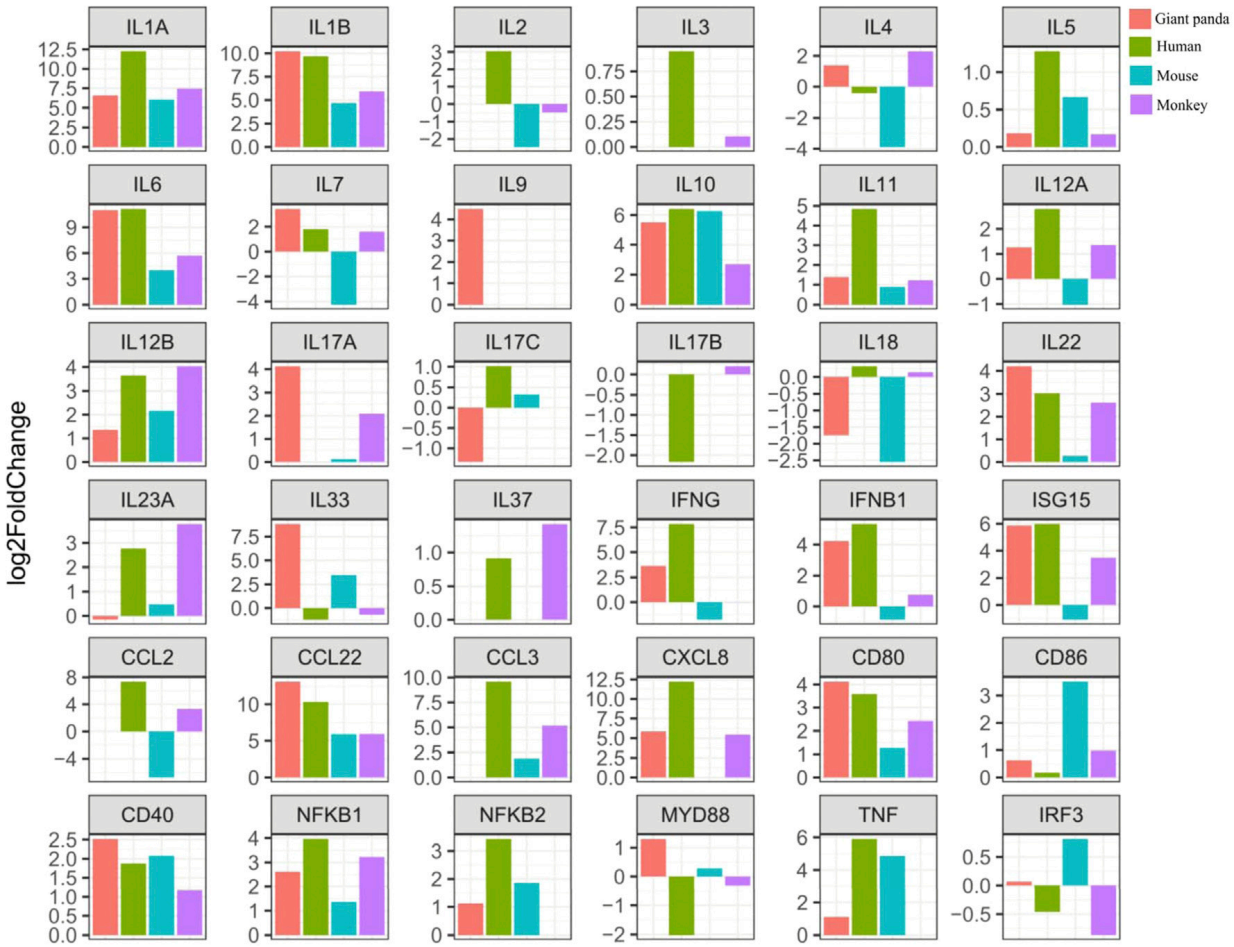
Expression tendency of selected gene expression among giant pandas, humans, mice, and monkeys.

**FIGURE 6:**
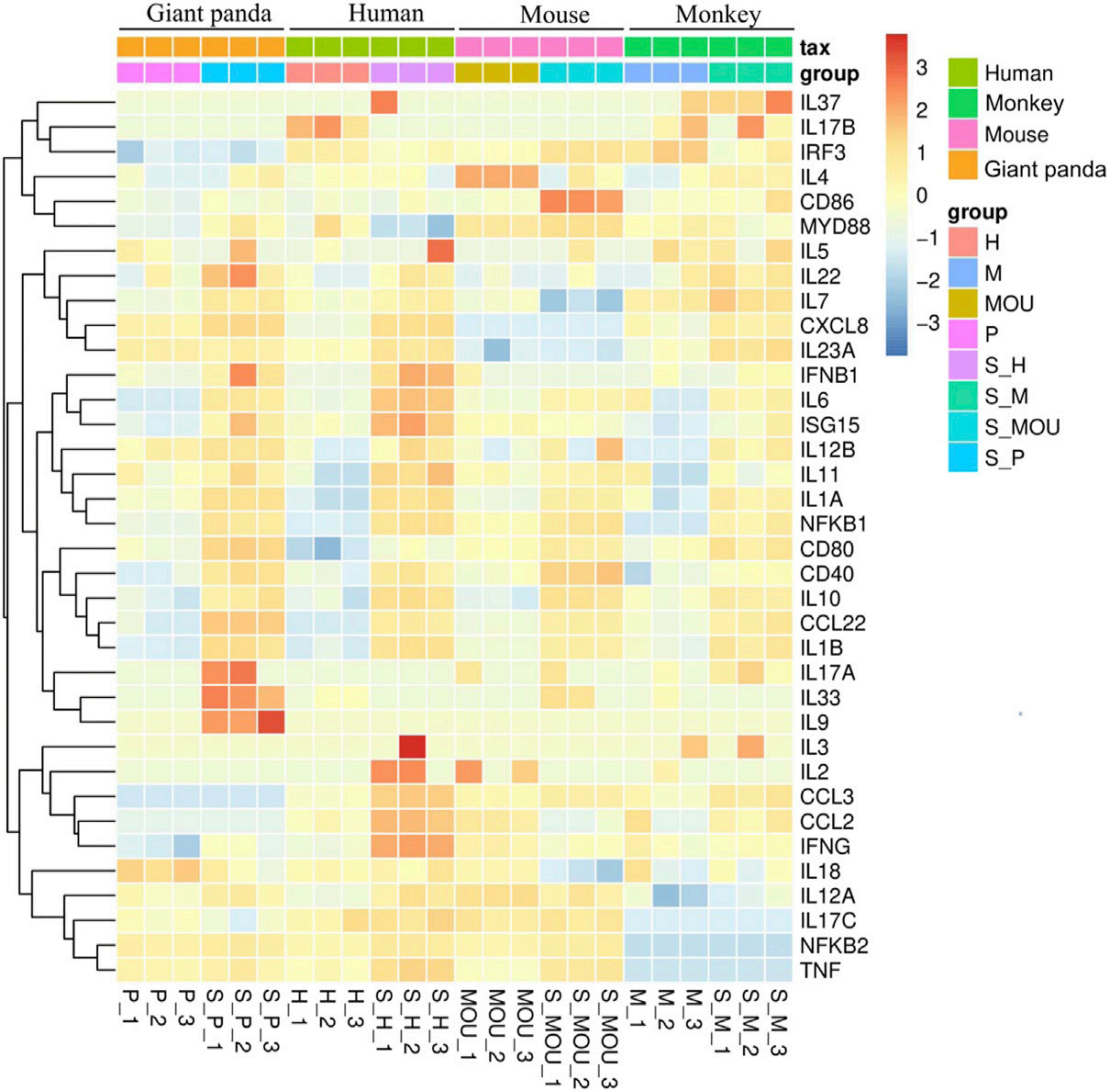
Heat map of representative gene expression across all samples from the four species.

**FIGURE 7:**
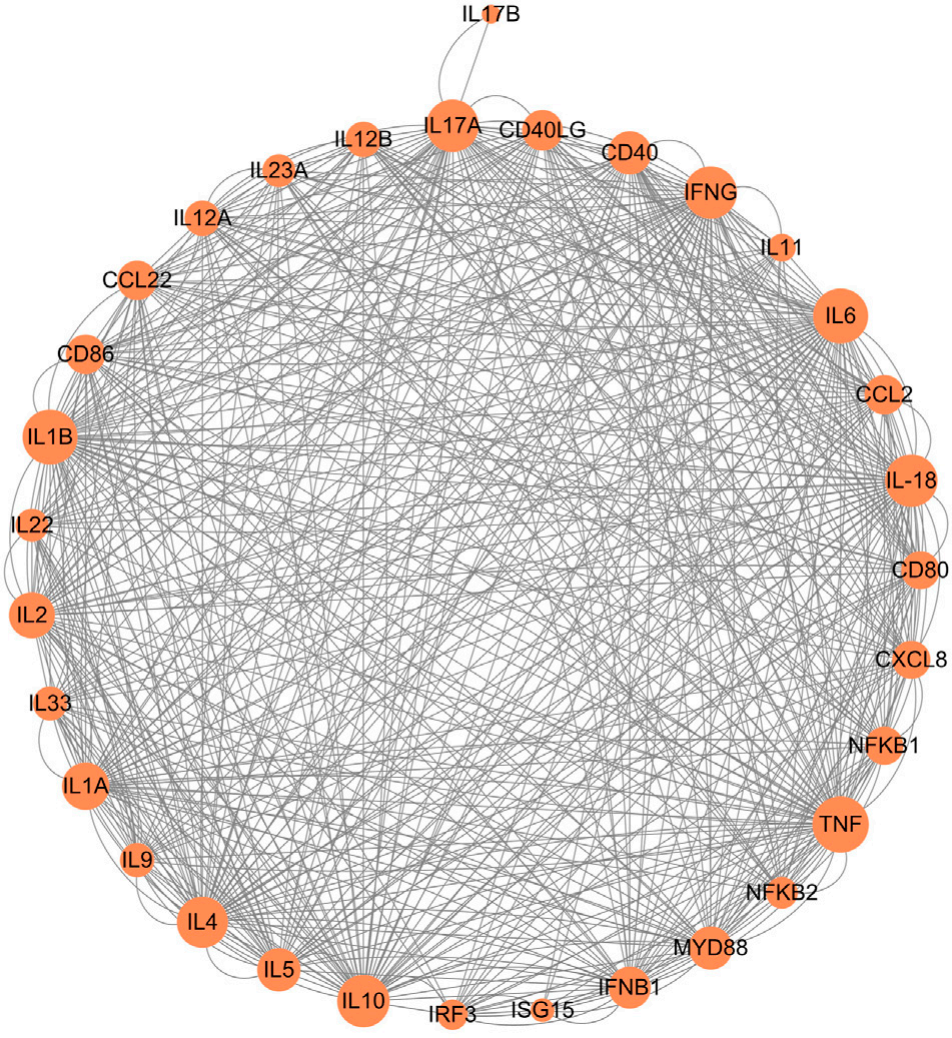
Network of protein–protein interaction analysis of immune-related genes.

**FIGURE 8:**
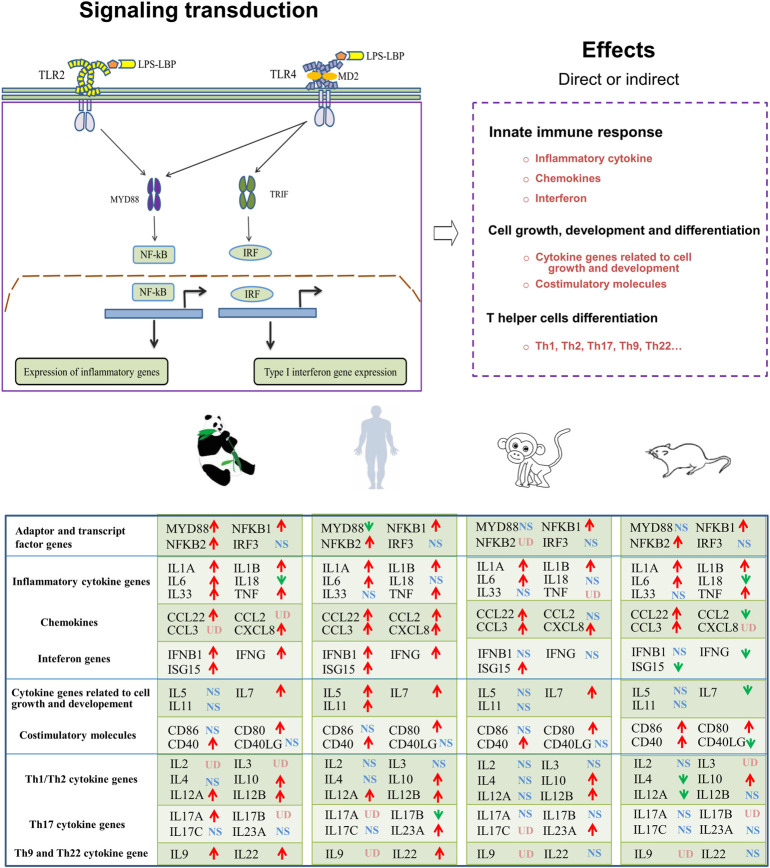
Graphic overview of the main signaling pathways and conclusions for the present study. LBP (LPS-binding protein); MD2 (myeloid differentiation factor 2); MyD88 (myeloid differentiation primary response 88); TRIF (TIR-domain-containing adapter-inducing interferon-β); IRF (IFN-regulatory factor); UD (undetected); NS (non-significant differential expression); ↑(significantly upregulated); ↓(significantly downregulated).

The Th9 and Th22 cytokine genes were also analyzed. IL-9, a characteristic cytokine produced by Th9 cells, acts as a regulator of a variety of hematopoietic cells. We found it was significantly upregulated in giant pandas, while it was undetectable in the gene lists of humans, monkeys, and mice. IL-22 was significantly upregulated in both giant pandas and humans, while it displayed no significant differential expression between the LPS-stimulated group and the control counterpart in both monkeys and mice (Figures 5, 6; Supplementary Table S1). These immune-related genes were converted into proteins by STRING, and the network diagram was plotted to show interactions among proteins. The interaction network showed that TNF, IFNG, IL-6, IL-1B, IL-18, and IL-17A had higher in-degree values and were hub genes (Figure 7). These results indicated species differences in immune responses to LPS (Figure 8).

## Discussion

As the first line of defense against infectious pathogens in mammals, the innate immune system plays a role in sensing and responding to PAMPs (Takeuchi and Akira, 2010). LPS, a PAMP of the Gram-negative bacteria, is predominantly recognized by the TLR4/MD2 receptor complex and activates inflammatory transcription factors mediated by NF-κB and interferon gene expression mediated through IRFs (Duan et al., 2022). It is important to appreciate that there are many differences in innate immune systems among species due to natural selection. *In vitro* activation of PBMCs by LPS is one method that has been widely applied for monitoring the immune system and identification of disease mechanisms (Slawinska et al., 2021; Gao et al., 2022).

Similar to the TLR4 from humans, mice, and monkeys, we found that the giant panda TLR4 protein also contains a number of LRR motifs and TIR homology domains, and its amino acid sequences showed 65%–77% similarity with the three other species. Although the TLR4 gene displays an orthology relationship across species (Roach et al., 2005), our transcriptomic profiles indicated that the LPS-stimulated gene expression, cytokine production and biological response were different to some extent. PBMCs activated by LPS lead to the innate immunity activation of monocytes and dendritic cells, featuring the inflammatory program mediated by NF-κB and interferon gene expression mediated by IRFs (Aksel and Akyüz, 2021). In our DEGs analyses, we observed that the NFKB1 showed increased expression across all four species, and NFKB2 was consistently upregulated in giant pandas, humans, and mice. In addition, the inflammatory cytokine genes IL-1A, IL-1B, and IL-6 showed increased expression across all four species, and TNF was consistently upregulated in giant pandas, humans, and mice. Moreover, the upregulated DEGs from giant pandas, as well as humans, monkeys, and mice, were found in the NF-κB signaling pathway. These data indicated that NF-κB is an LPS-responsive regulator of innate immune responses in giant pandas and other three species. Although the inflammatory cytokine IL-18 showed decreased expression both in giant pandas and mice, it showed no significant differential expression in humans and monkeys. This suggests that IL-18 was not the main inflammatory cytokine stimulated by LPS in PBMC across these four species at this time point. However, the other inflammatory cytokine, IL-33, was found to be significantly upregulated only in giant pandas, while it showed no significantly different expression in the other three species. IL-33 stimulates the early activation of Th2 responses (do Nascimento et al., 2022) and is also involved in the maturation of Th2 cells (Yu et al., 2015). The Th2 responses were activated after LPS-stimulated PBMCs in giant pandas at this time point but not in the other three species.

The expression pattern of chemokine analysis indicated that the expression trend of CCL22 was the same in the four tested species, and CXCL8 was significantly upregulated in giant pandas, humans, and monkeys. CCL2 and CCL3 were undetected in giant pandas, while they all showed increased expression in humans. These findings suggested that in addition to the similar chemokine expression patterns, giant pandas also have their own unique chemokine expression mode. Additionally, IFNG, IFNB1, and ISG15 were significantly upregulated after LPS activation in PBMCs from giant pandas and humans at the 6-h time point, indicating a similar time pattern of TLR4/LPS-activated IRF-mediated interferon responses in giant pandas and humans. In monkeys, although ISG15 showed increased expression, IFNG and IFNB1 showed no difference between the LPS-stimulated and -unstimulated group. In mice, IFNG and ISG15 exhibited significantly decreased expression. These data suggested that LPS-induced signaling pathways have different time patterns of response and sensitivity among these tested four species.

In addition to the monocytes and dendritic cells (DCs), lymphocytes are also a component of mammalian PBMCs (Li et al., 2021). TLRs-mediated activation and maturation of DCs play an important role in bridging innate immunity and cell-mediated immunity (Duan et al., 2022). TLR4 recognizes LPS, and DCs take up the antigen and present it to the CD4^+^ T cells for differentiation into Th type 1 (Th1) cells and Th2 cells (Iwasaki and Medzhitov, 2004). We observed that the costimulatory molecules CD80 and CD40 were upregulated across four species, indicating DC maturation and signal transduction were induced by LPS in the present study. We further checked the expression changes of Th1/Th2 cytokine genes (IL-2, IL-3, and IL-4), as IL-2 is secreted by Th1 cells, and IL-3 and IL-4 cytokines are secreted by Th2 cells. The secretion patterns of Th1/Th2 cytokine genes in giant pandas and monkeys were similar because IL-2 and IL-3 exhibited almost no expression, and IL-4 showed a trend of non-statistically significant increased expression after LPS stimulation at the 6-h time point. IL-2 and IL-3 were upregulated, and IL-4 was downregulated in humans, but the differences had no statistical significance. The expression of Th1/Th2 cytokine genes showed large differences in mice, as the IL-4 was significantly decreased and the IL-2 was non-statistically significantly downregulated at this time point. However, from the perspective of gene expression, these results were in agreement with the reported studies that Th1/Th2 cytokine gene activation from LPS-stimulated mammalian PBMCs is normally much less than inflammatory cytokines (Slawinska et al., 2021).

It has been reported that the anti-inflammatory cytokine gene IL-10 negatively regulates Th1 activation, whereas the pro-inflammatory cytokine gene IL-12 skews T cells toward Th1 immune responses (O’Garra and Murphy, 2009). Thus, the activated gene expression of IL-10 and the balanced expression of IL-12 play a protective role against excessive inflammation in the PBMCs. In our study, although IL-10 showed significantly increased expression in all four species, IL-12A showed significantly decreased expression in mice. IL-12A and IL-12B were significantly upregulated in giant pandas and humans at this time point. In addition, although the difference was not significant, IL-12A was upregulated, and IL-12B exhibited significantly increased expression in monkeys. These data indicated that the expressions of IL-10 and IL-12 were similar among giant pandas, humans and monkeys but diverse in mice after LPS stimulation at the 6-h time point.

Th17 cytokine gene IL-17A plays a pivotal role in various infectious diseases, inflammatory and autoimmune disorders, and cancer (Ge et al., 2020). Our results showed that it was significantly upregulated in the giant panda’s response to LPS; however, it showed no significant difference in either monkeys or mice and was undetected in humans. This suggests that IL-17A may play an important role in host defense against Gram-negative microbial pathogens in giant pandas at this time point. Moreover, IL-9, the characteristic cytokine produced by Th9 cells, only significantly increased expression in giant pandas, while it was undetected in humans, monkeys, and mice after LPS stimulation. In addition, IL-22, which can be produced by Th22, is a member of the IL-10 family of cytokines that mediate cellular inflammatory responses (Luo et al., 2021). The expression of IL-22 showed upregulated expression in both giant pandas and humans. These data indicated that Th9 and Th22 cells might be involved in the response to LPS in giant pandas at this time point.

In conclusion, the transcriptomic profiles of LPS-stimulated PBMCs at 6 h in giant pandas, humans, mice, and monkeys were successfully generated and comparatively analyzed in the present study. Our results indicated that in the giant panda, NF-κB is an LPS-responsive regulator of innate immune responses. Giant pandas and humans showed a similar time pattern regarding the TLR4/LPS-activated IRF-mediated interferon response. In addition, the secretion pattern of Th1/Th2 cytokine genes was similar between giant pandas and monkeys, and the expression patterns of IL-10 and IL-12 were alike among giant pandas, humans, and monkeys at the time point of 6 h *in vitro*. Finally, Th9, Th17, and Th22 cells might be involved in the response to LPS in giant pandas at this time point. Our data emphasized that the LPS-stimulated signaling pathway has different sensitivities and response timelines among various species. This study will be helpful for further understanding and investigating the TLRs signaling pathway and the immune system in giant pandas, which might open a new avenue for disease prevention and protection.

## Data Availability

The data presented in the study are deposited in the China National GeneBank DataBase (https://db.cngb.org/), accession number CNP0003545.

## Supplementary material

Supplementary Figure S1 Multiple alignments of the TLR4 amino acid sequence among giant pandas, humans, mice, and monkeys.

Supplementary Figure S2 Transcriptomic profiles of LPS-stimulated and -unstimulated PBMCs from mice by RNA-seq. **(A)** Flow diagram of the experimental design. **(B)** Volcano plot shows the DEGs by comparing the LPS-stimulated PBMCs and LPS-unstimulated PBMCs from mice at the time point of 6 h. Significantly differentially expressed genes were defined by both p-value (less than and equal to 0.05) and fold change (greater than and equal to 2). **(C)** GO and KEGG enrichments of upregulated DEGs for the group of LPS-stimulated PBMCs vs. LPS-unstimulated PBMCs from mice by RNA-seq. **(D)** GO and KEGG enrichments of downregulated DEGs for the group of LPS-stimulated PBMCs vs. LPS-unstimulated PBMCs from mice by RNA-seq.

Supplementary Figure S3 Transcriptomic profiles of LPS stimulated and -unstimulated PBMCs from monkeys by RNA-seq. **(A)** Flow diagram of the experimental design. **(B)** Volcano plot shows the DEGs by comparing the LPS-stimulated PBMCs and LPS-unstimulated PBMCs from rhesus monkeys at the time point of 6 hours. Significantly differentially expressed genes were defined by both p-value (less than and equal to 0.05) and fold change (greater than and equal to 2). **(C)** GO and KEGG enrichments of upregulated DEGs for the group of LPS-stimulated PBMCs vs. LPS-unstimulated PBMCs from rhesus monkeys by RNA-seq. **(D)** GO and KEGG enrichments of downregulated DEGs for the group of LPS-stimulated PBMCs vs. LPS-unstimulated PBMCs from rhesus monkeys by RNA-seq.
