# Supplementary figures and images for: Comparative transcriptome analyses of immune responses to LPS in peripheral blood mononuclear cells from the giant panda, human, mouse, and monkey
Source: Front Genet. 2023 Jan 6;13:1053655. doi: 10.3389/fgene.2022.1053655 (PMC9852843; doi:10.3389/fgene.2022.1053655)

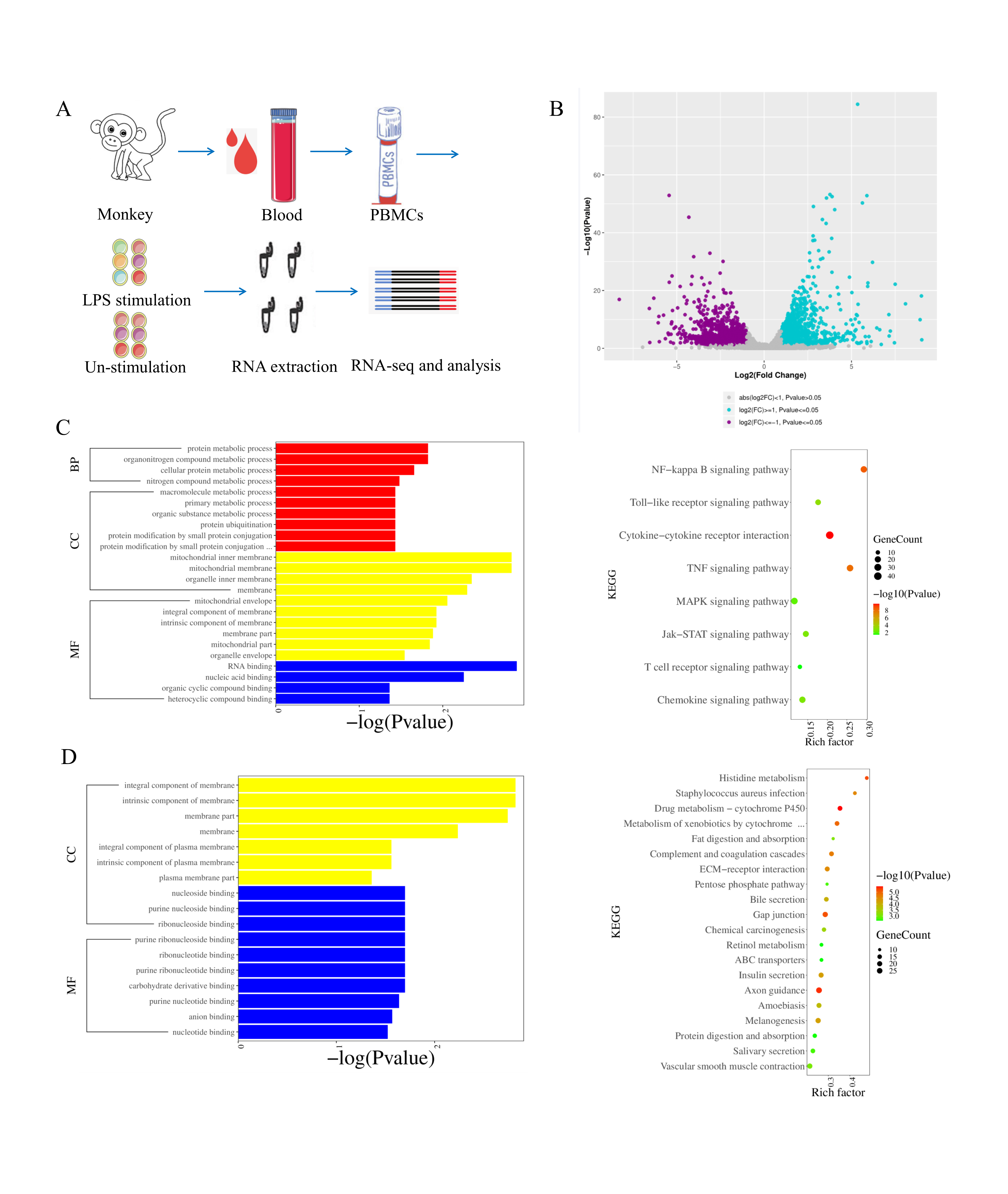

Supplement: Supplementary file 1 [file Image3.TIF]

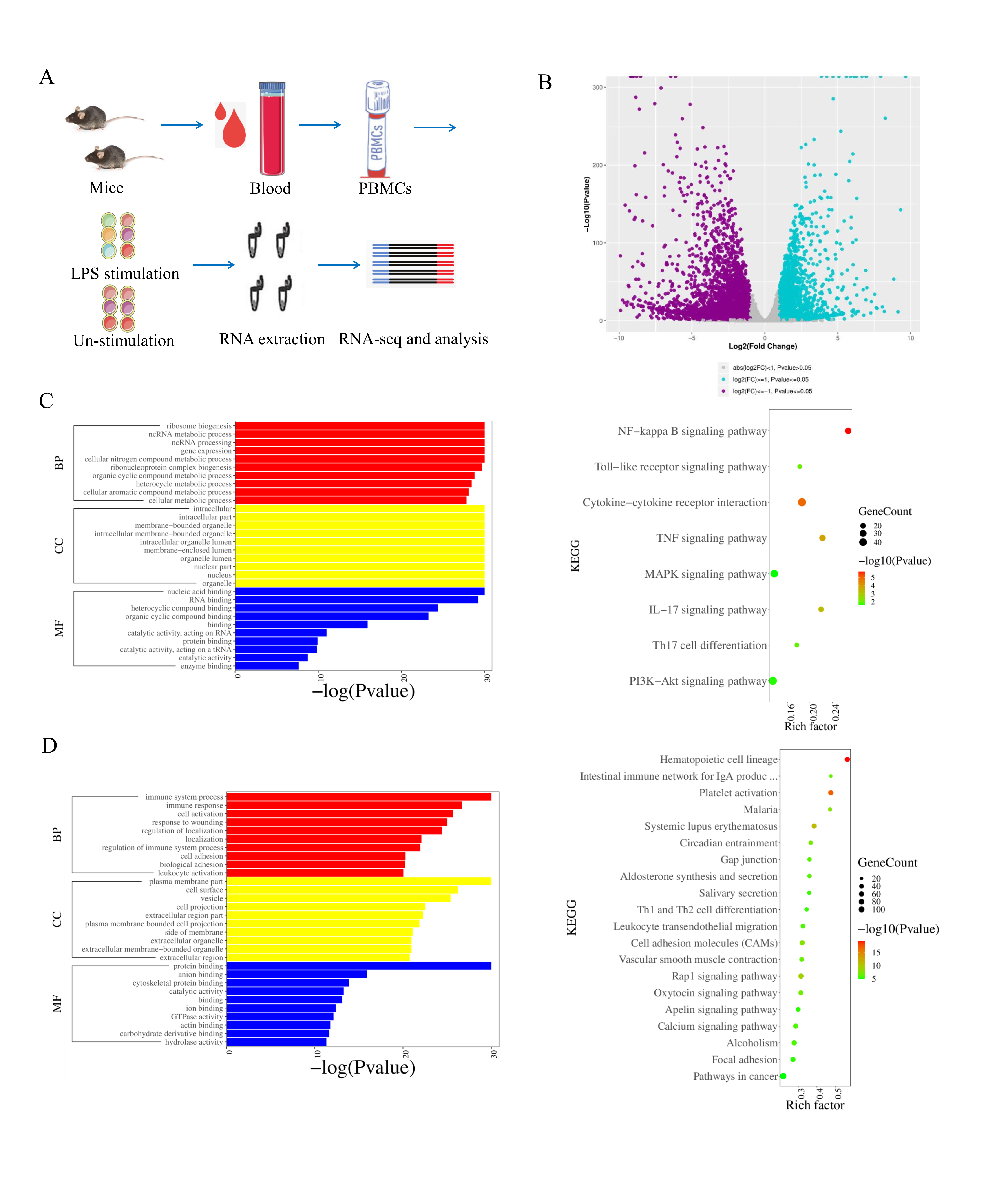

Supplement: Supplementary file 2 [file Image2.TIF]

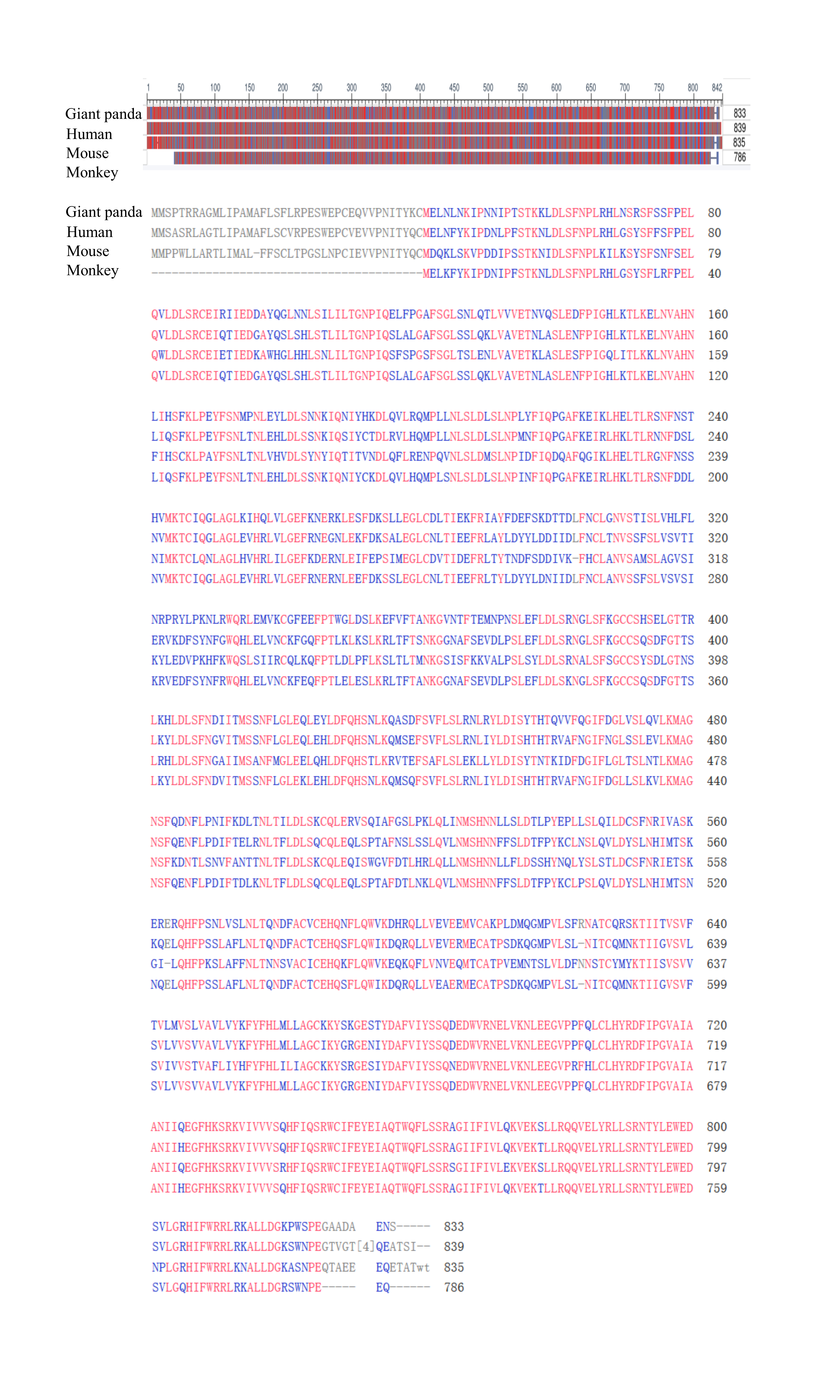

Supplement: Supplementary file 3 [file Image1.TIF]
